# Functional Evaluation of Neutralizing Antibodies Against Foot-and-Mouth Disease Virus Serotype O Using a Luciferase-Based Surrogate Neutralization Assay

**DOI:** 10.3390/v18091015

**Published:** 2026-09-14

**Authors:** Hyejin Kim, Dong-Wan Kim, Yeonrae Chae, Yerin Kim, Giyoun Cho, Ji-Hyeon Hwang, Yoon-Hee Lee, Jong-Hyeon Park, Sung-Han Park

**Affiliations:** Center for Foot-and-Mouth Disease Vaccine Research, Animal and Plant Quarantine Agency, 177, Hyeo-ksin 8-ro, Gimcheon-si 39660, Gyeongsangbuk-do, Republic of Korea; hyejin86@korea.kr (H.K.); dongwan12@korea.kr (D.-W.K.); codusfo1@korea.kr (Y.C.); yerin3531@korea.kr (Y.K.); libretto@korea.kr (G.C.); jihyeonh87@korea.kr (J.-H.H.); lyhee74@korea.kr (Y.-H.L.); parkjhvet@korea.kr (J.-H.P.)

**Keywords:** foot-and-mouth disease, serotype O, neutralizing antibodies, NanoBiT, virus-like particles, surrogate neutralization assay

## Abstract

Foot-and-mouth disease (FMD) is a highly contagious viral disease that seriously threatens livestock health. Protective immunity induced by vaccination is primarily associated with the generation of neutralizing antibodies; however, conventional virus neutralization tests are time-consuming and require the handling of live virus, limiting their suitability for rapid and repeated evaluation in routine settings. In this study, we established a surrogate neutralization assay for the functional evaluation of neutralizing antibody activity against FMD virus (FMDV) serotype O. The assay measures changes in luciferase-based luminescent signals generated following exposure of LgBiT-expressing cells to HiBiT-tagged virus-like particle (VLP) preparations. FMDV serotype O-derived VLPs exhibited stable capsid protein expression, assembly characteristics, and morphological integrity similar to those of virus particles, as confirmed by sucrose gradient fractionation and electron microscopy. In the presence of neutralizing antibodies, a reduction in luminescent signals was observed, enabling functional discrimination of antibody activity. The surrogate assay results showed a strong correlation with those of the conventional virus neutralization test for FMDV serotype O (*R*^2^ = 0.9068). This study demonstrates the feasibility of the surrogate neutralization assay for assessing the functional activity of neutralizing antibodies and its potential applicability to vaccine immunogenicity assessment and functional antibody analysis.

## 1. Introduction

Foot-and-mouth disease (FMD) is a highly transmissible viral disease affecting cloven-hoofed animals, including cattle, pigs, and goats. It is regarded as one of the most economically important transboundary animal diseases worldwide because it substantially impacts livestock production and international trade [1,2,3]. FMD virus (FMDV) is the etiological agent of FMD and is classified into seven immunologically distinct serotypes: O, A, C, Asia1, SAT1, SAT2, and SAT3 [4]. FMDV spreads rapidly through aerosol transmission and direct contact between infected and susceptible animals. Hence, effective vaccination and prompt disease control measures are essential for limiting viral dissemination during outbreaks [5,6,7]. Serotype O is recognized as the most widely distributed serotype worldwide and exhibits extensive genetic and antigenic variation [8,9]. This diversity highlights the importance of vaccine efficacy evaluation and antigenic matching for effective disease control [10].

Protective immunity induced by vaccination is essential for reducing clinical severity and viral replication following FMDV infection [1,6]. According to the WOAH Terrestrial Manual, broad serological surveillance may be required to identify animals that have developed antibodies following exposure to FMDV [11]. For this purpose, the virus neutralization test (VNT) and liquid-phase blocking ELISA (LPBE) are recommended for evaluating the antibody status against FMD in livestock [11,12]. Among these assays, virus-neutralizing antibodies are considered important serological indicators of protective immunity against FMDV infection and are widely used to evaluate vaccine-induced immune responses [11,13]. Additionally, neutralizing antibody titers correlate strongly with protection against FMD in vaccinated animals and are therefore commonly used as important indicators of vaccine efficacy [14]. However, the VNT requires the use of live virus and biosafety level 3 (BSL-3) containment facilities and typically takes 2–3 days to obtain results [11]. These limitations restrict its practical application for large-scale serological analysis and repeated testing, highlighting the need for alternative surrogate assays capable of safely and rapidly evaluating neutralizing antibody activity without the use of live virus [15].

Surrogate neutralization assays using pseudovirus- or virus-like particle (VLP)-based systems that can be applied in conventional laboratory settings have been actively developed in various fields of virology research [16]. Particularly, studies involving high-risk pathogens such as SARS-CoV-2 have combined luciferase-based reporter systems with pseudovirus or VLP platforms to quantitatively evaluate antibody-mediated inhibition of viral entry [17,18]. These luciferase-based assays offer several advantages, including high sensitivity, quantitative signal detection, rapid result acquisition, and suitability for high-throughput screening applications [19]. Moreover, these systems have shown strong correlations with conventional live-virus neutralization assays while allowing experiments to be performed under lower biosafety conditions [17,18,20].

Previous studies established a HiBiT-based NanoBiT platform for FMDV; however, these studies mainly focused on virus construction and the feasibility of NanoBiT signal detection [21]. The applicability of this platform for the functional evaluation of neutralizing antibody activity and vaccine immunogenicity assessment has not yet been fully investigated. Particularly, its applicability to serotype O, the most prevalent serotype circulating worldwide, is important for evaluating FMD vaccine efficacy.

Therefore, in this study, we investigated the applicability of a HiBiT-based NanoBiT reporter platform for functional neutralization analysis using FMDV serotype O VLPs. The performance of the assay was evaluated by comparison with the conventional VNT. Furthermore, we assessed its potential as a safe and efficient alternative platform for evaluating vaccine-induced immune responses and functional antibody activity against FMDV.

## 2. Materials and Methods

### 2.1. Cells

Human embryonic kidney 293T (HEK293T) cells (ATCC CRL-3216) were cultured in Dulbecco’s modified Eagle’s medium (DMEM, Gibco, Union City, NJ, USA) supplemented with 10% fetal bovine serum (FBS; Gibco, Grand Island, NY, USA) and 1% penicillin–streptomycin (Gibco) at 37 °C in a humidified incubator containing 5% CO_2_. HEK293T cells were used for the transient expression of HiBiT-tagged VLPs. A stable LgBiT-expressing LF-BK (LgBiT-LF-BK) cell line previously established in our laboratory was used for the NanoBiT-based neutralization assay. The generation and characterization of the LgBiT-LF-BK cell line have been described previously [21].

### 2.2. Construction of HiBiT-Tagged Plasmids

**Figure 1:**
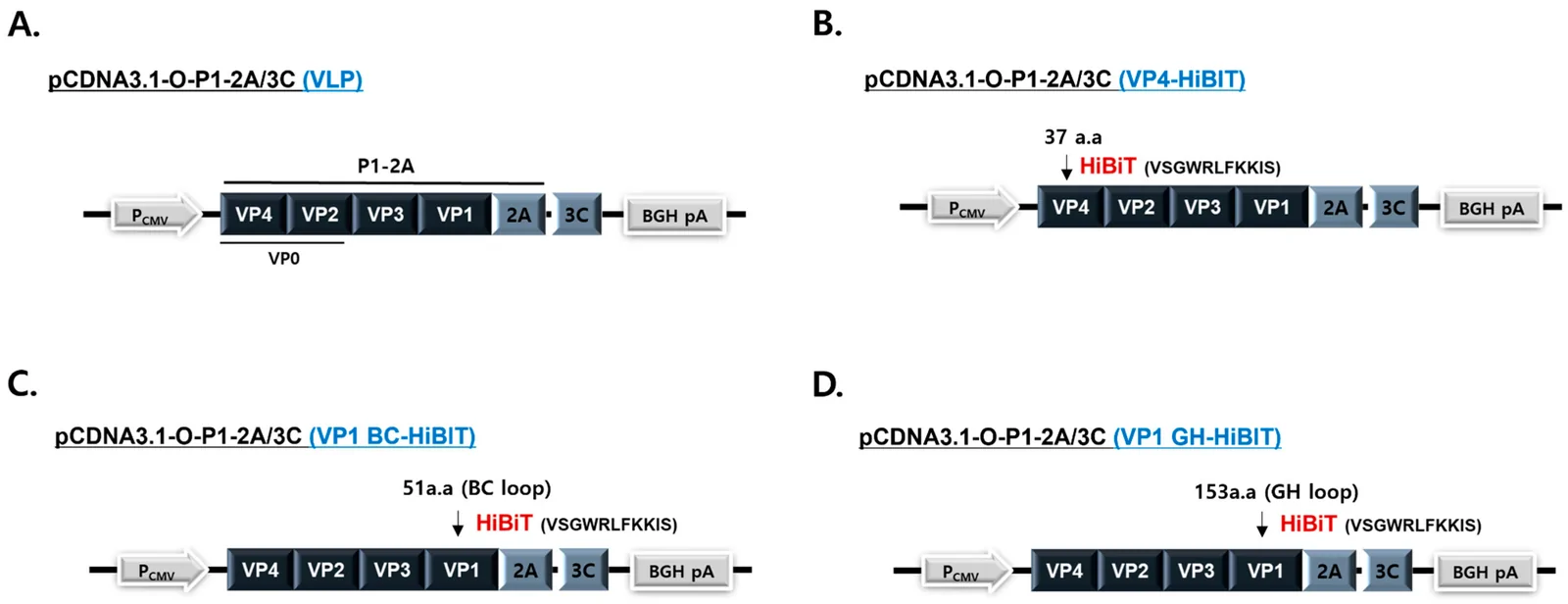
Schematic representation of HiBiT-tagged FMDV serotype O VLP expression constructs: (**A**) Schematic representation of the control plasmid without a HiBiT tag (pcDNA3.1-O-P1-2A/3C and VLP). (**B**–**D**) Plasmids containing the HiBiT tag inserted at amino acid position 37 of VP4 ((**B**) VP4-HiBiT), position 51 within the VP1 BC loop ((**C**) VP1 BC-HiBiT), and position 153 within the VP1 GH loop ((**D**) VP1 GH-HiBiT). The HiBiT insertion sites are indicated by arrows.

The P1-2A/3C coding sequence of FMDV serotype O/PanAsia-2 (GenBank accession no. GU384682.1) was cloned into the CMV promoter-driven pcDNA3.1 expression vector to generate the VLP expression plasmid (Figure 1A). The 3C protease carries an L127P substitution that reduces cytotoxicity while maintaining proteolytic activity [22]. To construct HiBiT-tagged VLP expression plasmids, the HiBiT peptide sequence (VSGWRLFKKIS), flanked by GSSG linker sequences (GSSG VSGWRLFKKISGSSG), was inserted after amino acid 37 of VP4, amino acid 51 within the VP1 BC loop, and amino acid 153 within the VP1 GH loop. The resulting plasmids were designated VP4-HiBiT, VP1 BC-HiBiT, and VP1 GH-HiBiT, respectively (Figure 1B–D). Each expression plasmid was transiently transfected into HEK293T cells using the FuGENE HD transfection reagent (Promega, Madison, WI, USA) according to the manufacturer’s instructions. Following incubation for 48 h, both cell lysates and culture supernatants were collected and used for subsequent analyses.

### 2.3. Expression of HiBiT-Tagged VLPs

The expression of HiBiT-tagged VLPs was confirmed by Western blot, luciferase, and transmission electron microscopy (TEM; H-7100FA; Hitachi, Tokyo, Japan) analyses. For Western blot analysis, HEK293T cells were co-transfected with each HiBiT-tagged VLP expression plasmid and an LgBiT expression plasmid. Cell lysates and culture supernatants were collected 48 h after transfection. Protein samples were separated by SDS-PAGE, transferred onto PVDF membranes (Bio-Rad Laboratories, Hercules, CA, USA), and probed with anti-HiBiT (Promega, Madison, WI, USA), anti-LgBiT (Promega), or anti-FMDV VP2 antibodies (MEDIAN Diagnostics, Chuncheon-si, Republic of Korea). HRP-conjugated secondary antibodies (Millipore, Billerica, MA, USA) were used for detection, and immunoreactive bands were visualized using an enhanced chemiluminescence (ECL) substrate (Amersham, Buckinghamshire, UK). For the NanoBiT reporter activity assay, culture supernatants containing HiBiT-tagged VLPs were incubated with stable LgBiT-expressing LF-BK cells, and luminescence signals were measured using the Nano-Glo Luciferase Assay system (Promega) according to the manufacturer’s instructions. HiBiT-tagged VLPs were concentrated and purified as previously described [23]. Purified VLPs were separated by ultracentrifugation through a 15–45% sucrose density gradient, and the absorbance of each fraction was measured at 280 nm. The morphology of purified VLPs was examined by TEM following negative staining with 1% uranyl acetate.

### 2.4. Animal Serum

All animal experiments and serum collection procedures were approved by the Institutional Animal Care and Use Committee (IACUC) of the Animal and Plant Quarantine Agency (APQA), Republic of Korea (Approval No. 2024-860).

To evaluate the ability of the NanoBiT-VNT to discriminate different levels of neutralizing antibodies against FMDV serotype O, serum samples were collected from five 2-month-old pigs vaccinated intramuscularly with a single 2 mL dose of a bivalent FMD vaccine containing the O/Boeun and A/Yeoncheon strains. O/Boeun and the O/PanAsia-2 assay targets belong to different lineages (Ind-2001e and PanAsia-2, respectively) within the serotype O ME-SA topotype, whereas A/Yeoncheon belongs to serotype A (ASIA/Sea-97). The vaccinated sera showed measurable neutralizing activity against O/PanAsia-2 and were therefore used to compare the two assays.

Serum samples were collected at 0, 28, and 56 days post-vaccination (dpv), yielding a total of 15 serum samples. After clotting, serum was separated by centrifugation at 3000× *g* for 15 min at 4 °C and stored at −70 °C until analysis. Based on the conventional VNT results against FMDV serotype O, the serum samples were classified into three groups (*n* = 5 samples per group): VN titer ≤ 0.9 log10, VN titer > 0.9 to ≤1.5 log10, and VN titer > 1.5 to ≤2.0 log10.

To evaluate the correlation between the conventional VNT and NanoBiT-VNT, 66 serum samples were collected exclusively from pigs vaccinated with a bivalent FMD vaccine containing the O/Boeun and A/Yeoncheon strains (with no history of natural FMDV infection). According to the distribution of the conventional VNT titers against FMDV serotype O, the serum samples were categorized into four groups: VN titer < 0.9 log10 (*n* = 15), VN titer 0.9–1.51 log10 (*n* = 11), VN titer 1.65–2.41 log10 (*n* = 19), and VN titer ≥ 2.5 log10 (*n* = 21). These serum samples were used to evaluate the correlation between neutralizing antibody titers determined by the conventional VNT and NanoBiT-VNT.

### 2.5. ELISA and Conventional VNT

Serum samples were heat-inactivated at 56 °C for 30 min before testing. Antibodies against FMDV serotype O structural proteins were measured using the VDPro FMDV Type O Ab b-ELISA Kit (MEDIAN Diagnostics, Chuncheon, Republic of Korea) according to the manufacturer’s instructions. Results were interpreted according to the manufacturer’s recommended criteria. The VNT against FMDV serotype O/ME-SA/PanAsia-2 was performed according to the WOAH Manual of Diagnostic Tests and Vaccines for Terrestrial Animals [11]. Briefly, two-fold serially diluted sera were incubated with 100 TCID50 of the O/ME-SA/PanAsia-2 virus for 1 h at 37 °C in 96-well plates. LF-BK cell suspension was then added to each well, and the plates were incubated at 37 °C for 72 h.

The virus neutralization titers were calculated using the Spearman–Kärber method [24,25] in accordance with the standard operating procedures of the World Reference Laboratory for Foot-and-Mouth Disease [26].

### 2.6. NanoBiT-VNT

The NanoBiT-VNT was performed as previously described [23]. Briefly, stable LgBiT-expressing LF-BK cells were seeded into 96-well plates at a density of 2 × 10^4^ cells/well. HiBiT-tagged serotype O VLPs were incubated with two-fold serially diluted serum samples (1:16 to 1:512, starting at 1:16) for 1 h at 37 °C and then transferred to stable LgBiT-expressing LF-BK cells. Luciferase activity was measured using a Nano-Glo Luciferase Assay System (Promega, Madison, WI, USA), and neutralizing antibody titers were determined based on the percentage inhibition (PI) of luciferase activity as previously described [23]. All measurements were performed in technical triplicate.

### 2.7. Statistical Analysis

Data are presented as the mean ± standard error of the mean (SEM). Statistical analyses were performed using GraphPad Prism software (version 8.4.3; GraphPad Software, San Diego, CA, USA). Differences between groups were analyzed using one- or two-way analysis of variance (ANOVA) followed by Tukey’s or Sidak’s multiple-comparison test, as appropriate. Differences were considered statistically significant at *p* < 0.05.

## 3. Results

### 3.1. Construction of HiBiT-Tagged FMDV Serotype O VLPs

A previous study demonstrated that insertion of a HiBiT tag into the VP1 GH loop (153 a.a.) of FMDV serotype Asia1 enabled NanoBiT-based luciferase signal detection [21]. To identify an optimal insertion site for HiBiT tagging in FMDV serotype O, three constructs were designed by inserting the HiBiT sequence into the VP4 (37 a.a.), VP1 BC loop (51 a.a.), or VP1 GH loop (153 a.a.) within the structural protein-coding region (Figure 1). These regions were selected because they are surface-exposed or structurally permissive sites that could potentially accommodate peptide insertion without disrupting capsid assembly [27,28]. The schematic plasmid maps illustrate the HiBiT insertion sites in the serotype O constructs, providing the basis for subsequent expression and functional analyses. A non-tagged VLP construct was included as a control. These constructs were subsequently used to evaluate protein expression, NanoBiT-mediated luminescence signals, and their suitability for functional neutralization analysis.

### 3.2. Evaluation of HiBiT Expression and NanoBiT Reporter Activity of FMDV Serotype O Constructs

**Figure 2:**
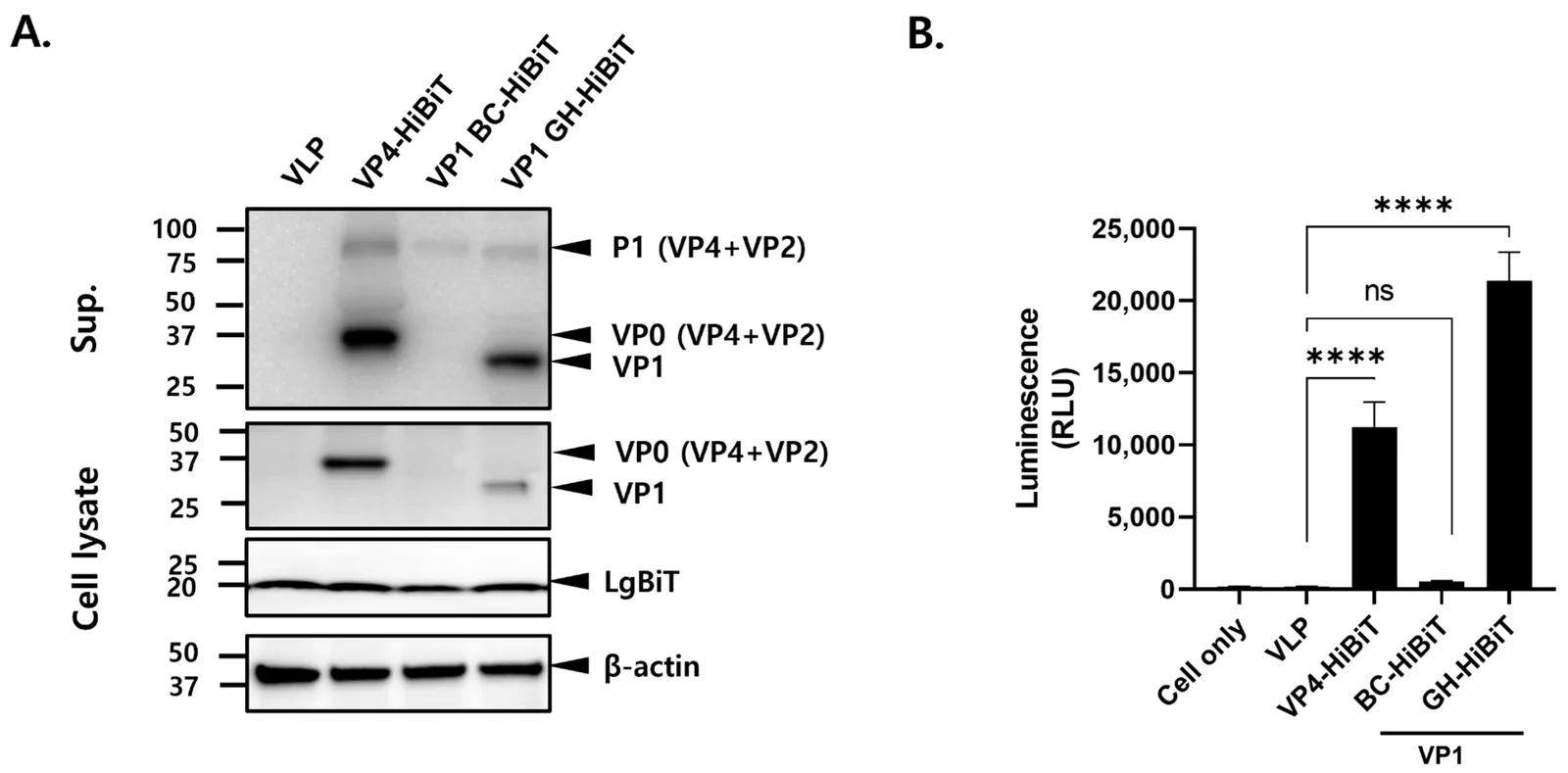
Expression and luciferase activity of HiBiT-tagged FMDV serotype O VLPs: (**A**) Protein expression of HiBiT-tagged VLPs in cell lysates and culture supernatants of HEK293T cells co-transfected with HiBiT-tagged VLP and LgBiT expression plasmids. (**B**) NanoBiT reporter activity measured after incubation of culture supernatants containing HiBiT-tagged VLPs with stable LgBiT-expressing LF-BK cells. Data are presented as the mean ± SEM from three independent experiments (*n* = 3). Statistical significance was analyzed using one-way ANOVA followed by a multiple-comparison test (ns, not significant; ****, *p* < 0.0001).

To evaluate the expression characteristics of the HiBiT-tagged constructs, HEK293T cells were co-transfected with HiBiT-tagged VLP and LgBiT expression plasmids. Cell lysates and culture supernatants were collected 48 h post-transfection and analyzed using Western blotting with an LgBiT- or HiBiT-specific antibody (Figure 2A). Distinct HiBiT-specific bands were detected in both cell lysates and culture supernatants from the VP4-HiBiT and VP1 GH-HiBiT constructs, whereas relatively weak signals were observed for the VP1 BC-HiBiT construct. For both VP4-HiBiT and VP1 GH-HiBiT, signal intensities were stronger in culture supernatants than in cell lysates. These results indicate that the expression and/or accessibility of the HiBiT tag may differ depending on the insertion site.

To evaluate NanoBiT reporter activity, culture supernatants containing HiBiT-tagged VLPs were incubated with stable LgBiT-expressing cells, and luminescence signals were subsequently measured (Figure 2B). Both the VP4- and VP1 GH-HiBiT constructs generated significantly higher luminescence signals than those of the control, whereas the VP1 BC-HiBiT construct produced signals comparable to background levels. Among the constructs tested, the VP1 GH-HiBiT construct exhibited the highest luminescence signals, while the VP4-HiBiT construct also generated substantial luminescence. In contrast, little or no reporter activity was detected with the VP1 BC-HiBiT construct. These findings indicate that insertion of the HiBiT tag into either VP4 or the VP1 GH loop enables effective NanoBiT-based signal generation following incubation of HiBiT-tagged VLP preparations with stable LgBiT-expressing cells.

### 3.3. Characterization of HiBiT-Tagged FMDV Serotype O VLPs

**Figure 3:**
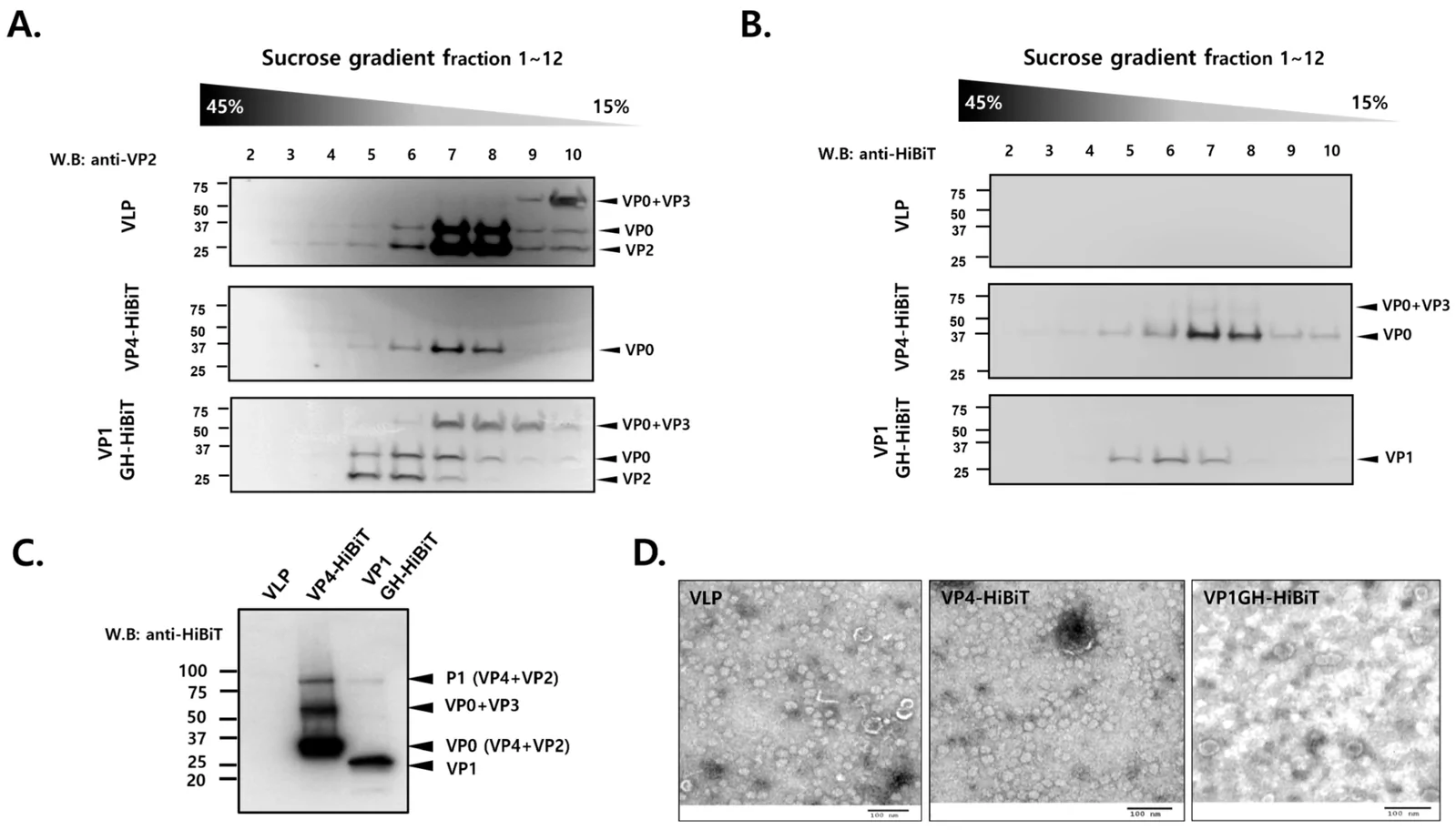
Purification and characterization of HiBiT-tagged FMDV serotype O VLPs: (**A**,**B**) Distribution of capsid proteins and HiBiT-tagged proteins in sucrose density gradient fractions, detected using anti-VP2 and anti-HiBiT antibodies, respectively. (**C**) Detection of HiBiT-tagged proteins in pooled peak fractions using an anti-HiBiT antibody. (**D**) Representative transmission electron microscopy (TEM) images of purified VLPs, VP4-HiBiT VLPs, and VP1 GH-HiBiT VLPs. Scale bars = 100 nm.

To characterize the formation of HiBiT-tagged VLPs, purified particles were subjected to sucrose gradient ultracentrifugation, and individual fractions were analyzed by Western blotting (Figure 3A,B). Using an anti-VP2 antibody, VP0 and VP2 proteins were detected in fractions corresponding to the VLP-containing regions in both the VP4- and VP1 GH-HiBiT preparations (Figure 3A). In addition, Western blot analysis using an anti-HiBiT antibody demonstrated that HiBiT-tagged proteins were detected in the same fractions containing VLP-associated proteins (Figure 3B). This distribution pattern indicated the presence of HiBiT-tagged proteins within VLP-associated fractions and provided the basis for selecting fractions for subsequent analyses. Western blot analysis of the pooled fractions further confirmed the presence of both VP4- and VP1 GH-HiBiT proteins (Figure 3C), indicating that HiBiT-tagged proteins were successfully recovered from the selected sucrose gradient fractions. The purified VLPs were subsequently examined by TEM. TEM analysis confirmed the presence of particles exhibiting VLP morphology in the purified preparations. Together, these findings provide supporting evidence for the association of HiBiT-tagged proteins with VLP-containing preparations.

### 3.4. Evaluation of the VP4-HiBiT VLP-Based NanoBiT Neutralization Assay Using Porcine Sera

To evaluate the ability of the VP4-HiBiT VLP-based NanoBiT assay to detect neutralizing antibodies, sera collected from pigs vaccinated with a bivalent FMDV vaccine containing the O/Boeun and A/Yeoncheon vaccine strains were analyzed (*n* = 15). Based on the results of the conventional VNT against FMDV serotype O, the sera were classified into three groups: negative (VN titer ≤ 0.9 log10), positive serum 1 (0.9 < VN titer ≤ 1.5 log10), and positive serum 2 (1.5 < VN titer ≤ 2.0 log10).

**Figure 4:**
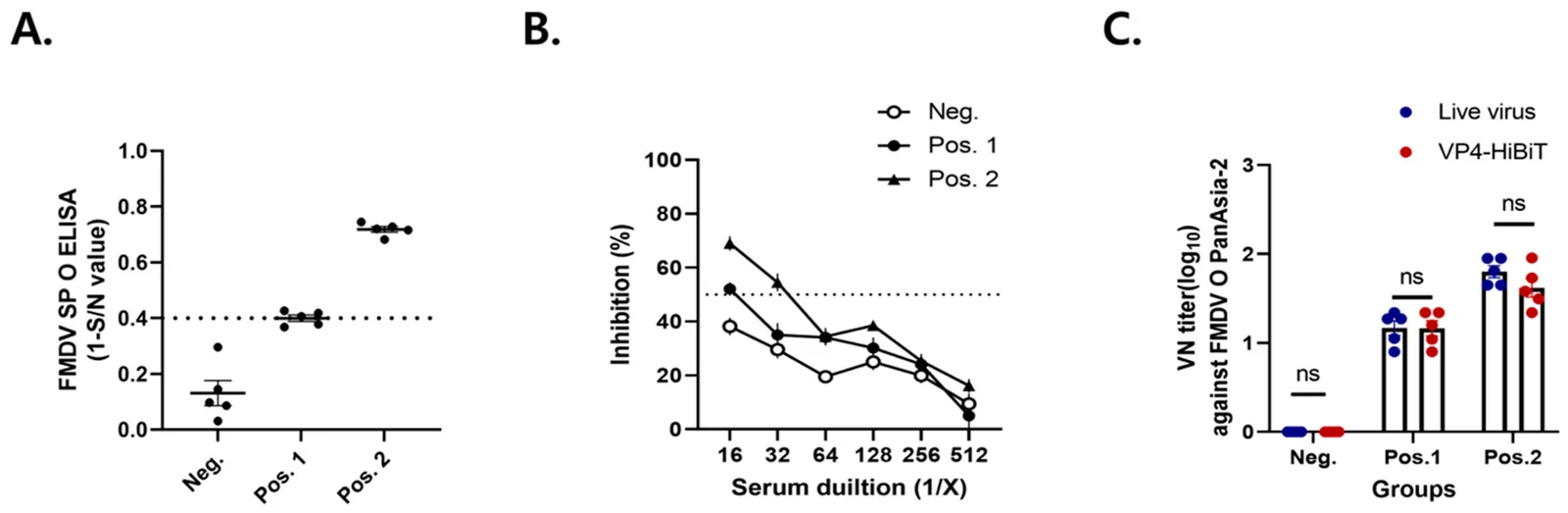
Evaluation of the NanoBiT-VNT using porcine serum samples: (**A**) FMDV serotype O SP ELISA results for negative sera (Neg.) and two groups of positive sera (Pos. 1 and Pos. 2). The dashed line indicates the ELISA cutoff value. (**B**) Neutralization of VP4-HiBiT VLPs by negative and positive porcine sera. Serum samples were serially diluted from 1:16 to 1:512, and percent inhibition was determined using the NanoBiT assay. The dashed line indicates the 50% inhibition threshold. (**C**) Comparison of neutralizing antibody titers determined by the conventional VNT and the NanoBiT-VNT using porcine serum samples. Statistical significance was analyzed using two-way ANOVA followed by Sidak’s multiple-comparison test. ns, not significant.

ELISA analysis showed that most sera in the negative group were below the positivity threshold, whereas sera in both positive groups exceeded the cutoff value (Figure 4A). In addition, the positive serum 2 group exhibited higher ELISA responses than those of the positive serum 1 group, consistent with the VNT results. NanoBiT neutralization curves demonstrated a progressive decrease in inhibition activity (%) with increasing serum dilution in all groups (Figure 4B). All sera in the positive serum 2 group maintained inhibition rates above 50% at a dilution of 1:32, whereas all sera in the positive serum 1 group remained above the 50% inhibition threshold at a dilution of 1:16. Conversely, sera in the negative group generally failed to achieve 50% inhibition across the dilution series. Comparison of neutralizing antibody titers quantified by the NanoBiT assay and the conventional live-virus VNT revealed no significant differences between the two assays within each serum group (Figure 4C). Moreover, both assays displayed similar trends in neutralizing antibody levels among the three serum groups. These findings suggest that the VP4-HiBiT VLP-based NanoBiT assay can effectively reflect neutralizing antibody responses against FMDV serotype O and has potential as a surrogate approach for neutralizing antibody assessment.

### 3.5. Correlation Between the VP4-HiBiT VLP-Based NanoBiT Assay and Conventional VNT

**Figure 5:**
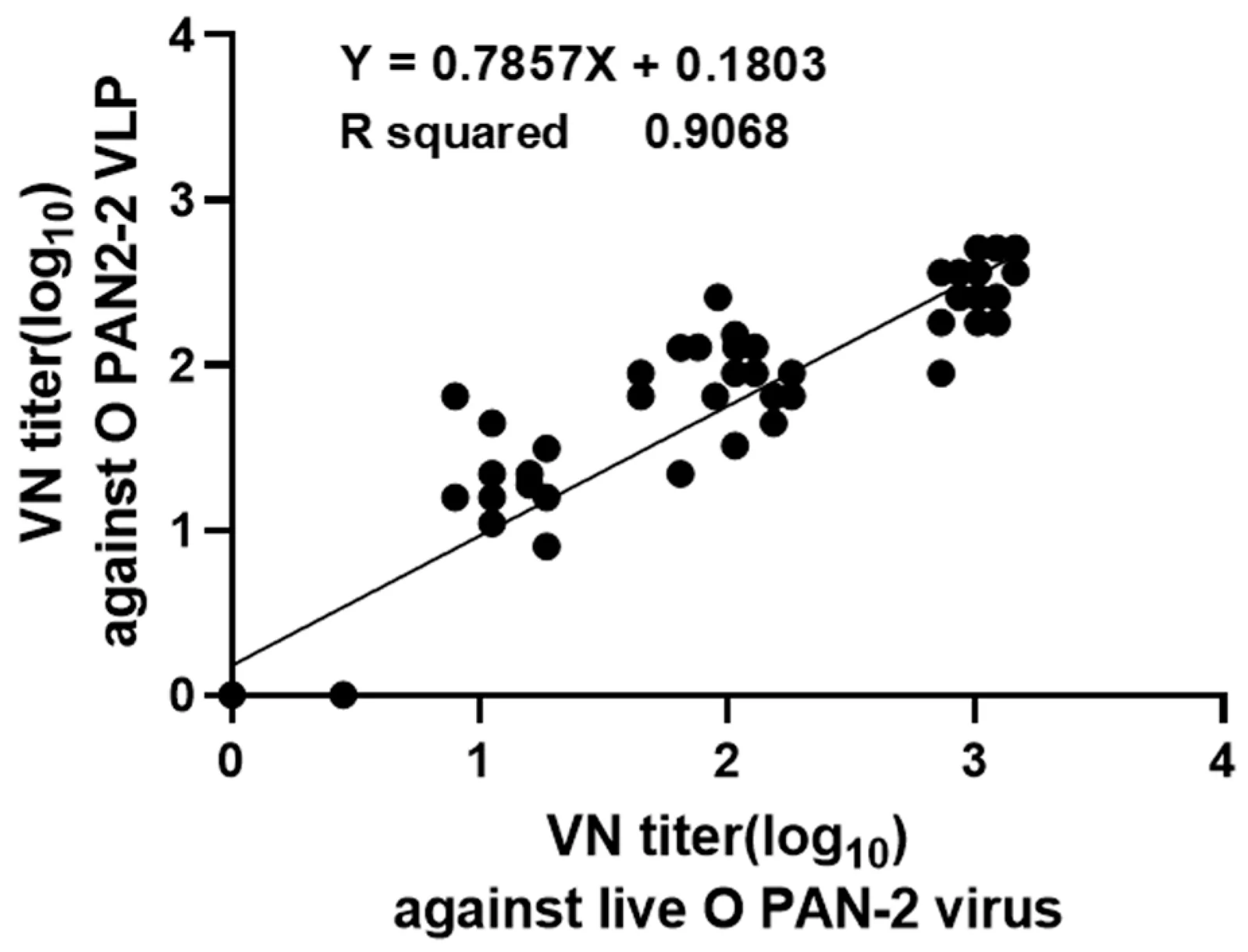
Correlation between neutralizing antibody titers determined by the conventional virus neutralization test (VNT) and the VP4-HiBiT VLP-based NanoBiT-VNT. Neutralizing antibody titers against FMDV serotype O were measured using the conventional VNT and the VP4-HiBiT VLP-based NanoBiT-VNT in 66 porcine serum samples. The correlation between the titers quantified by the two assays was analyzed by linear regression.

To further evaluate the VP4-HiBiT VLP-based NanoBiT assay, neutralizing antibody titers determined from 66 porcine serum samples were compared with those by the conventional live-virus VNT (Figure 5). Serum samples exhibiting low neutralizing antibody titers in the conventional VNT generally showed low NanoBiT-derived titers, whereas those with high VNT titers exhibited correspondingly high NanoBiT neutralization titers. This relationship was consistently observed across the entire range of antibody responses analyzed. Neutralizing antibody titers measured by the two assays showed a strong positive correlation, with a coefficient of determination (*R*^2^) of 0.9068 (Figure 5). However, the regression slope was below unity (0.7857), indicating that the relationship between the two assays was not strictly proportional and that NanoBiT-derived titers tended to increase less steeply than conventional VNT titers, particularly at the higher end of the titer range. Nevertheless, these findings indicate that neutralizing antibody titers measured using the VP4-HiBiT VLP-based NanoBiT assay are strongly associated with those determined by the conventional live-virus VNT and support its potential as a surrogate platform for evaluating vaccine-induced neutralizing antibody responses.

## 4. Discussion

Neutralizing antibody responses are widely regarded as one of the most important indicators for assessing the immunogenicity and protective efficacy of FMD vaccines [11]. The conventional VNT employing live FMDV remains the reference method for measuring functional neutralizing antibodies and is routinely used for vaccine evaluation and serological studies [11]. Nonetheless, the assay requires the handling of infectious virus under high-containment laboratory conditions and is relatively labor-intensive and time-consuming, limiting its broader application for large-scale serological screening [11]. To address these limitations, alternative neutralization assays based on reporter viruses, pseudoviruses, and VLPs have been increasingly explored in various viral systems [29,30,31]. These approaches provide safer and more rapid platforms for measuring neutralizing antibody responses while maintaining a strong correlation with conventional neutralization assays.

A previous study demonstrated the feasibility of a NanoBiT-based neutralization platform through the insertion of a HiBiT tag into the VP1 GH loop of FMDV serotype Asia1 VLPs [23]. That study primarily focused on reporter signal generation and proof-of-concept evaluation of the platform. In contrast, the present study extended the NanoBiT system to FMDV serotype O, the most prevalent serotype responsible for the majority of FMD outbreaks worldwide [26]. Furthermore, we evaluated the applicability of this platform for measuring neutralizing antibody activity using porcine serum samples.

In the present study, VP4, the VP1 BC loop, and the VP1 GH loop were evaluated as potential insertion sites for the HiBiT tag. Both the VP4-HiBiT and VP1 GH-HiBiT constructs exhibited detectable protein expression and reporter activity, whereas the VP1 BC-HiBiT construct showed relatively low expression and reporter signals. The VP1 BC and GH loops are hypervariable regions located on the surface of the FMDV capsid and contain important B- and T-cell epitopes involved in host immune recognition [32,33,34,35]. In addition, the VP1 GH loop contains the conserved RGD motif required for integrin receptor binding and virus entry [36]. Therefore, inserting a heterologous peptide such as HiBiT into these functionally important regions may alter the local structural environment or accessibility of the inserted tag, which may have contributed to the differences in reporter activity observed among the constructs.

Although the VP1 GH–HiBiT construct generated higher raw luminescence signals than the VP4–HiBiT construct (Figure 2), it was less suitable for use in the VNT. VP1 GH–HiBiT VLPs produced a high background signal and elevated levels of non-specific inhibition, thereby limiting the reliable assessment of serum neutralizing activity. Consistent with this reduced assay performance, sucrose density gradient centrifugation and transmission electron microscopy (TEM) analyses showed that insertion of the HiBiT tag into the VP1 GH loop impaired proper VLP assembly, resulting in heterogeneous and partially disrupted particles. These structural abnormalities may have produced unassembled capsid proteins or disrupted particle fragments in which the HiBiT peptide was readily accessible, thereby increasing non-specific luminescence and interfering with the accurate measurement of neutralization. In contrast, VP4–HiBiT VLPs retained a more intact particle morphology and exhibited a reproducible, dilution-dependent neutralization response. These findings indicate that reporter signal intensity alone does not necessarily reflect the structural integrity or functional suitability of VLPs for neutralization assays. Because preservation of the native antigenic structure is essential for measuring antibody-mediated neutralization, the VP4 region was considered a more suitable HiBiT insertion site for the serotype O VLP-based NanoBiT system. The present findings also indicate that the VP1 GH loop, which was successfully utilized in the previously reported serotype Asia1-based NanoBiT platform, may not necessarily represent the optimal insertion site across all FMDV serotypes [23]. FMDV serotypes exhibit considerable differences in amino acid composition and capsid architecture, which may influence the structural environment surrounding the inserted HiBiT tag [37]. These serotype-dependent differences may affect reporter activity, particle assembly, and the functional performance of the resulting VLPs. Therefore, when NanoBiT-based platforms are developed for different FMDV serotypes, the insertion site should be optimized to achieve an appropriate balance between reporter signal intensity and preservation of VLP structure.

Furthermore, preservation of intact capsid structure is important for maintaining the native presentation of neutralizing epitopes, as capsid dissociation can induce conformational changes that alter their recognition by neutralizing antibodies [38]. In the icosahedral capsid of FMDV, VP1–VP3 form the external antigenic surface, with the VP1 GH loop constituting a surface-exposed neutralizing site, whereas VP4 is located on the internal face of the capsid [39]. Therefore, insertion of the small 11-amino-acid HiBiT tag into VP4 may be less likely to interfere directly with surface-exposed neutralizing epitopes than insertion into the VP1 GH loop. This structural arrangement may help preserve the antigenic presentation of the VLPs and may partly explain the more reliable neutralization response observed with the VP4–HiBiT construct.

The VP4-HiBiT VLP-based NanoBiT assay effectively distinguished negative from positive sera and provided a dilution-dependent inhibition profile. Based on the 50% inhibition cutoff, sera from the positive 2 group remained positive at a 1:32 dilution, whereas sera from the positive 1 group remained positive up to a 1:16 dilution. These findings indicate that the assay can differentiate between varying levels of neutralizing antibody responses among serum samples. Furthermore, evaluation using a panel of 66 porcine serum samples demonstrated a strong correlation between the NanoBiT assay and the conventional live-virus VNT (*R*^2^ = 0.9068). However, the regression slope was below unity (0.7857), indicating that the relationship between the two assays was not strictly proportional. In particular, NanoBiT-derived titers tended to be relatively lower than conventional VNT titers at the higher end of the response range, suggesting a compression of the NanoBiT-derived titer range. Therefore, the strong correlation between the two assays should not be interpreted as numerical equivalence of the measured titers.

While the conventional VNT reflects the complete viral infection process, including viral replication following cell entry, the NanoBiT assay measures reporter activity generated following exposure of LgBiT-expressing cells to HiBiT-tagged VLP preparations and does not involve productive viral replication. Several analytical and biological factors may therefore contribute to the observed proportional difference, including differences in the effective dynamic ranges of the two assays, characteristics of the luminescence-based reporter response, VLP input and cellular interaction efficiency, and the methods used to derive neutralization titers. The relative compression observed at higher titers may also reflect limitations in the upper response range of the NanoBiT assay; however, the present data do not allow the contribution of individual factors, including reporter signal saturation, to be determined. Further analytical validation across a broader range of antibody titers will be required to characterize this relationship. Nevertheless, the strong correlation observed in this study indicates that neutralizing antibody responses measured by the NanoBiT assay are strongly associated with those determined by the conventional VNT.

The NanoBiT-VNT may also offer practical advantages over the conventional VNT in terms of assay time and sample throughput. The conventional VNT generally requires 48–72 h of incubation for the evaluation of virus-induced cytopathic effects and must be performed in an appropriate high-containment laboratory using infectious FMDV. In contrast, the NanoBiT-VNT can be completed within several hours and is compatible with a multiwell format and a luminescence-capable microplate reader. The absence of infectious virus reduces the need for high-containment infrastructure during routine testing and may decrease the associated labor and operating costs. Although a formal cost analysis was not performed in the present study, these characteristics support the potential use of the NanoBiT-VNT for high-throughput serological screening.

Serotype specificity is another important consideration for the application of this assay to field samples. Because the present NanoBiT-VNT uses serotype O VLPs displaying serotype-specific capsid epitopes, it is expected to predominantly detect neutralizing antibodies against serotype O FMDV. However, the extent of cross-reactivity with antibodies against other FMDV serotypes was not evaluated in the present study. Further testing using well-characterized sera against heterologous serotypes, including serotypes A and Asia1, will therefore be required to determine the analytical specificity of the assay. Although sucrose density gradient fractionation, Western blotting, and TEM provided supporting evidence for the association of HiBiT-tagged proteins with VLP-containing preparations, the present study did not directly demonstrate that the NanoBiT signal was derived predominantly from the cellular entry of intact VP4-HiBiT VLPs. Therefore, potential contributions from soluble HiBiT-containing proteins, partially assembled particles, or disrupted particle material cannot be completely excluded. Further mechanistic studies directly linking particle integrity, cellular entry, and NanoBiT signal generation will be required to establish the biological basis of the assay readout.

Despite these promising results, several technical and manufacturing challenges should be addressed before the VLP-based NanoBiT-VNT platform can be implemented in routine diagnostic laboratories. Reproducible production of intact VLPs at a sufficient yield will require standardized manufacturing procedures and rigorous batch-to-batch quality control. In particular, the proportion of intact particles, reporter activity, and background luminescence should be monitored as critical quality attributes because variations in particle assembly may directly affect assay performance. Although Western blotting and TEM confirmed capsid protein expression and VLP formation (Figure 3), further studies should assess sample purity and particle-size distribution using SDS-PAGE and quantitative particle-size analysis. Luminescence measurements in the present study were performed in technical triplicate. While the present findings demonstrate the feasibility of the assay, further validation will be required to determine intra- and inter-assay precision, expressed as CVs, and to assess consistency among different VLP production batches. Further analytical validation and standardization of the platform, including characterization of the assay response range across low- and high-titer sera, will therefore be required before routine diagnostic application.

The present study demonstrates that the location of HiBiT insertion is a critical determinant of both VLP structural integrity and functional assay performance. Although insertion into the VP1 GH loop generated a strong luminescence signal, the resulting structural abnormalities and non-specific assay responses limited its suitability for neutralization testing. In contrast, VP4–HiBiT VLPs maintained comparatively intact particle morphology and enabled dilution-dependent measurement of serum neutralizing activity. The strong correlation with the conventional VNT supports the potential application of this platform as a non-infectious alternative for assessing neutralizing antibody responses against FMDV serotype O. Further optimization of VLP production, purification, stability, and inter-laboratory reproducibility will be required before the assay can be adopted for routine diagnostic or large-scale serological applications.

## 5. Conclusions

The present study demonstrated the feasibility of a VP4-HiBiT VLP-based NanoBiT assay for assessing neutralizing antibody responses against FMDV serotype O using porcine serum samples. The strong correlation with the conventional VNT supports the potential of this platform as a surrogate approach for functional antibody assessment. Further analytical validation, including assessment of assay precision, reproducibility, and analytical range, as well as evaluation using additional FMDV serotypes and well-characterized serum panels, will be required to establish its broader utility. These findings support the feasibility of the VP4-HiBiT/NanoBiT system as a surrogate platform for neutralizing antibody assessment, although further mechanistic studies are required to establish the extent to which the assay signal specifically reflects intact VLP-dependent cellular entry.

## Data Availability

The data supporting the findings of this study are available from the corresponding author upon reasonable request. For further information or inquiries regarding the data, please contact the corresponding author.

## Abbreviations

The following abbreviations are used in this manuscript:

ANOVA	Analysis of variance
APQA	Animal and Plant Quarantine Agency
BSL-3	Biosafety level 3
DMEM	Dulbecco’s modified Eagle’s medium
ECL	Enhanced chemiluminescence
FMD	Foot-and-mouth disease
FMDV	Foot-and-mouth disease virus
HEK293T	Human embryonic kidney 293T
IACUC	Institutional Animal Care and Use Committee
LPBE	Liquid-phase blocking ELISA
PI	Percentage inhibition
SEM	Standard error of the mean
TEM	Transmission electron microscopy
VLP	Virus-like particle
VNT	Virus neutralization test
WRLFMD	World Reference Laboratory for Foot-and-Mouth Disease
